# The Dilemma of Surgical Timing in Acute Aortic Valve Endocarditis: Does Early Surgery Improve Risks or Prognosis?

**DOI:** 10.3390/jcdd12040153

**Published:** 2025-04-11

**Authors:** Michele D’Alonzo, Lorenzo Di Bacco, Antonio Fiore, Massimo Baudo, Francesca Zanin, Chiara Baldelli, Cyrus Moini, Thierry Folliguet, Claudio Muneretto

**Affiliations:** 1 Cardiac Surgery Unit, University of Brescia, “Spedali Civili” Hospital, 25124 Brescia, Italy; l.dibacco@unibs.it (L.D.B.); f.zanin@unibs.it (F.Z.); claudio.muneretto@unibs.it (C.M.); 2 Cardiac Surgery Unit, Hôpital “Henri Mondor—Assistance Publique Hôpîtaux de Paris”, 94100 Créteil, France; antonio.fiore@aphp.fr (A.F.); thierry.folliguet@aphp.fr (T.F.); 3 Department of Cardiac Surgery Research, Lankenau Institute for Medical Research, Main Line Health, Wynnewood, PA 19096, USA; massimo.baudo@icloud.com; 4 School of Medicine and Surgery, University of Brescia, 25124 Brescia, Italy; c.baldelli@studenti.unibs.it; 5 Department of Cardiology, Groupe Hospitalier Sud Ile de France, 77000 Melun, France; cyrus.moini@ghsif.fr

**Keywords:** infective endocarditis, aortic valve, homograft, early treatment, timing, antibiotics

## Abstract

Background: Acute aortic valve infective endocarditis (IE) presents a critical surgical timing dilemma. This study investigates whether early surgical intervention (within seven days of targeted antibiotic therapy initiation) affects mortality and clinical outcomes compared to delayed/conventional surgery. Methods: A retrospective, multicenter analysis of 204 patients with aortic IE was conducted, excluding emergency cases requiring immediate intervention. Patients were stratified into EARLY (≤7 days) and LATE (>7 days) surgical groups. Primary endpoints included in-hospital mortality and major adverse events, while secondary endpoints assessed long-term survival, recurrence, and reintervention rates. Results: No significant differences in in-hospital mortality were observed between groups (16% in both). The LATE group exhibited a trend toward increased permanent pacemaker implantation (16% vs. 8.2%; *p* = 0.100) and a higher incidence of postoperative atrial fibrillation (36% vs. 24%, *p* = 0.048). Infective endocarditis recurrence and long-term survival did not significantly differ between groups. Predictors of one-year mortality included chronic kidney disease, annular abscess, extracorporeal membrane oxygenation (ECMO) use, and prolonged mechanical ventilation. Conclusions: These findings suggest that early surgery, following a short course of antibiotics, does not compromise outcomes nor increase recurrence risk, challenging the conventional preference for delayed intervention in non-emergency IE cases.

## 1. Introduction

Infective endocarditis (IE) is a serious condition characterized by an infection affecting various cardiac structures to differing degrees of severity, including the endocardium, native or prosthetic heart valves, and implanted cardiac devices. IE remains a relatively uncommon condition, with an annual occurrence of approximately 3–10 cases per 100,000 individuals [1]; however, it is widely believed that the incidence of infective endocarditis is increasing and remains underreported [2].

The evolution of IE exhibits significant variability and dynamic clinical progression. This evolving landscape is driven by the aging population, the growing prevalence of multidrug-resistant organisms, advancements in diagnostic technologies, and the expanding role of more invasive surgical treatments.

To address the growing complexity of IE management, the ESC guidelines introduced the concept of the “Endocarditis Team” in 2015 [3]. This emphasizes the need for a multidisciplinary approach to target optimal treatment strategy for IE. The diverse manifestations of infective endocarditis demand careful decision-making by the Endocarditis Team. A key challenge is determining the optimal timing for surgery, which is crucial for minimizing neurological complications, controlling infection progression, and improving postoperative outcomes and survival rates.

To date, the choice of optimal timing for surgery on IE is primarily based on observational studies, due to the challenges of designing randomized trials in such a complex context. The 2023 ESC guidelines [4] precisely outline which cases require emergency surgery within 24 h; urgent surgery within 3–5 days, or delayed surgery after a short course of antibiotics with close clinical and echocardiographic monitoring. Nevertheless, no consensus exists on the optimal timing of surgical treatment during the active phase of infection because of a lack of evidence-based data.

A significant concern is the risk of recurrence, which often requires high-risk reoperation. For decades, early cardiac surgery without completing sufficient targeted antibiotic therapy has been thought to increase recurrence risk in both native and prosthetic valves [5,6].

A limitation in the existing literature is the tendency to consider infective endocarditis as a generalized condition, treating aortic and mitral cases as if they share identical characteristics. The primary focus of this study is to focus exclusively on aortic endocarditis, with the inclusion of patients with multivalvular involvement only when the primary site of infection is aortic. This study aims to evaluate the hypothesis that delaying intervention until at least seven days after initiating targeted antibiotic therapy may improve outcomes following cardiac surgery.

## 2. Materials and Methods

This retrospective, observational, multicenter study aimed to evaluate the outcomes of patients undergoing cardiac surgery for acute infective endocarditis, with a specific focus on the aortic valve, including both native and prosthetic valves.

### 2.1. Patients

The study enrolled patients affected by aortic IE between January 2015 and June 2024. The study population was selected from the institutional databases of two tertiary institutions: Spedali Civili Hospital—Brescia, Italy, and Henri Mondor Hospital—Créteil, France. Preoperative clinical and demographic data were collected, including age, gender, major comorbidities, and symptoms at hospital admission. Diagnosis of IE was based on clinical findings (fever, inflammatory syndromes), laboratory tests (blood cultures, culture or 16S rRNA amplicon-sequencing performed on vegetation or cardiac tissue, Coxiella burnetii or Bartonella species serologies, leucocytosis, levels of C-reactive protein and procalcitonin), transthoracic/transoesophageal echocardiography and multi-imaging techniques including Computed tomography scan (CT), positron emission tomography (PET), magnetic resonance imaging (MRI), and intraoperative findings. Targeted therapy is defined as an antibiotic regimen guided by blood culture results and susceptibility testing (antibiogram). Emergency surgery (<24 h between diagnosis and treatment), patients < 18 years of age, those without an infectious focus on the aortic valve, and those who did not undergo surgery were excluded from the study.

### 2.2. Definitions, Endpoints and Follow-Up

From the entire study population of 223 patients, emergency interventions (19 patients) were excluded, as these individuals could not delay surgery to complete the antibiotic course. The remaining cohort was then categorized into two groups: the *EARLY* group, comprising patients who underwent surgery within 7 days of initiating antibiotic therapy, and the *LATE* group, including those treated surgically after at least 7 days of antibiotic therapy. The primary endpoint is to compare in-hospital mortality and the incidence of major adverse events between the two groups. Secondary endpoints focus on long-term outcomes: initially evaluating mortality alone, followed by analyzing a composite endpoint that includes death, reintervention (for any cause, either infectious or non-infectious), stroke, and permanent pacemaker implantation. Finally, the study aims to identify variables associated with one-year mortality following cardiac surgery for infective endocarditis through logistic regression analysis. Follow-up was conducted up to August 2024 (mean follow-up: 637 days) by phone interview or clinical examination, when possible.

### 2.3. Statistical Analysis

Distribution normality was analyzed with the Kolmogorov–Smirnov test. Continuous variables were compared using independent Student’s *t*-test with a two-tailed distribution if normally distributed. For non-normally distributed variables, the Mann–Whitney U-test was used. Categorical variables were compared using the chi-square χ^2^ or Fisher’s exact test as needed. Survival differences between the two groups were represented and compared using the Kaplan–Meier method and log-rank tests. A logistic regression model was employed to identify variables correlated to the one-year mortality in the overall population. The effect size of the variables was estimated by calculating the odds ratio (OR) and 95% confidence interval (CI). A *p*-value ≤ 0.05 was considered statistically significant. Microsoft Office Excel (Microsoft, Redmond, WA, USA) was used for data extraction, and all analyses were performed in R, version 4.3.1 (R Software for Statistical Computing, Vienna, Austria) within RStudio (2024.09.1). The R packages used were “survival”, “survminer”, “dplyr”, “gtsummary”, “bloom”, and “ggplot2”.

## 3. Results

After applying the inclusion and exclusion criteria, we analyzed the results of 204 patients undergoing surgical intervention for aortic endocarditis. Those patients were divided into EARLY (within 7 days of appropriate antibiotic therapy, 97 patients) and LATE (after 7 days, 107 patients) intervention groups.

### 3.1. Preoperative

Table 1 summarizes the baseline characteristics. The median surgical delay was significantly longer in the LATE group (15.0 days vs. 3.0 days, *p* < 0.001). No significant differences were observed between groups in terms of age, gender distribution, body surface area (BSA), or body mass index (BMI). Rates of major comorbidities were similar. Clinical features, such as cardiogenic shock, fever, and nosocomial infections, showed no statistical difference. The prevalence of high NYHA functional class (III–IV) was higher in the EARLY group (36% vs. 26%, *p* = 0.13), though not statistically significant. Similarly, other complications, including splenic abscesses and previous cerebrovascular events, were comparable between groups.

Table 2 highlights similar echocardiographic, anatomical, and laboratory characteristics between the groups, except for a higher prevalence of large vegetations in the EARLY group (*p* = 0.02) and slightly elevated white blood cell counts (*p* = 0.049). Other parameters, including valve involvement, abscesses, hemoglobin, albumin, and LVEF, showed no significant differences.

### 3.2. Causative Microorganism

**Figure 1:**
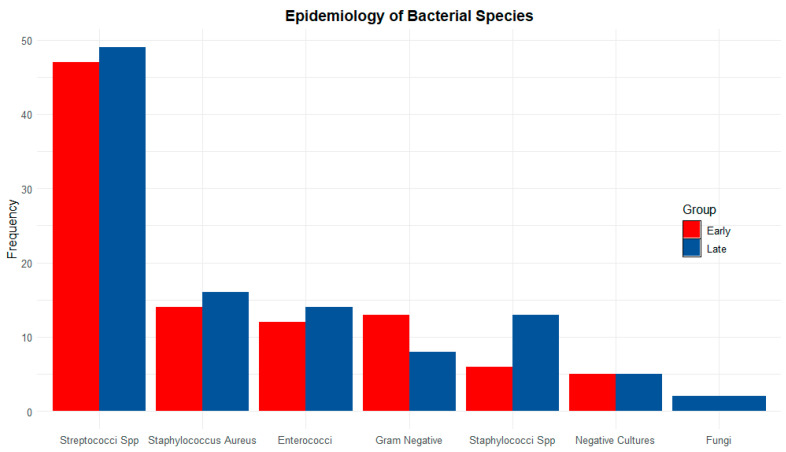
Frequency of microorganisms isolated from in-hospital cultures, grouped by surgical timing. Red: early surgery; Blue: late surgery; Spp: species.

Figure 1 shows the distribution of microorganisms isolated from in-hospital cultures, grouped according to the timing of surgical intervention. Notably, the analysis reveals no significant differences between species and groups, indicating that the isolated microorganism is not correlated with the timing of surgery.

### 3.3. Surgical Technique

In 92% of cases (188 patients), surgical AVR was performed, with a biological valve used in most cases (178/204, 85%). A mechanical valve was implanted in only 9 patients (4.4%). No significant differences were observed between groups in the use of biological or mechanical prostheses. The Bentall procedure was performed in 6.9% of patients, with no difference between groups. Rates of additional mitral surgery or coronary artery bypass grafting and operative times were also comparable between the two populations. Surgical approaches are listed in detail in Table 3.

### 3.4. Perioperative (In-Hospital Course)

Table 4 outlines postoperative complications and outcomes, including the primary endpoint of in-hospital mortality. In-hospital mortality was similar in both the EARLY and LATE groups, with each experiencing a 16% rate (*p* = 1.000). The LATE group had a higher, though not statistically significant, need for a permanent pacemaker compared to the EARLY group. (EARLY 8.2% versus LATE 16%, *p* = 0.100). No significant differences were observed between the two groups regarding need of mechanical support with ECMO, prolonged invasive ventilation, or postoperative stroke. A significant increase in the incidence of postoperative atrial fibrillation was observed in the LATE group (EARLY 24% vs. LATE 36%%, *p* = 0.048). Acute renal failure rates were comparable between groups, with no substantial differences in the distribution of renal impairment stages according to KDIGO classification.

### 3.5. Follow-Up (Out-Hospital Course)

Table 5 presents post-hospitalization outcomes, excluding patients who died during the main hospitalization. The relapse of infective endocarditis was similar between the groups (14% in EARLY vs. 11% in LATE, *p* = 0.6). New cerebrovascular events were rare and similar across groups (2.9%). Mortality at the last follow-up showed no significant difference (19% in EARLY vs. 14% in LATE, *p* = 0.5).

**Figure 2:**
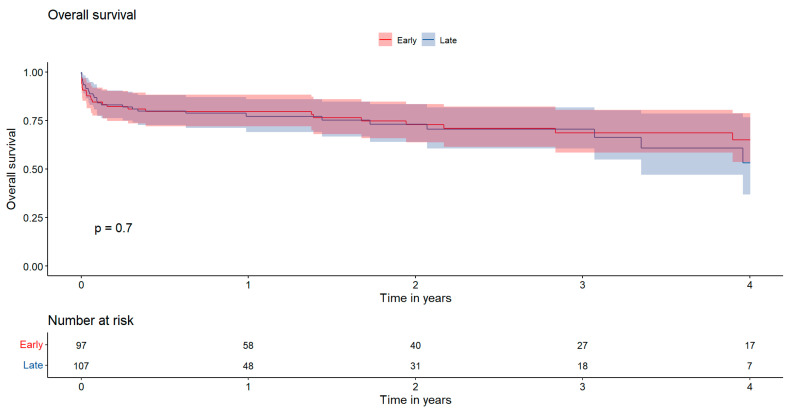
Presents the 4-year overall survival rates, which were 65.0 ± 12.5% in the EARLY group and 53.2 ± 19.4% in the LATE group, showing no statistically significant difference between the two groups (*p* = 0.70).

Figure 2 Kaplan–Meier survival curves illustrating overall survival. The *x*-axis represents the time in years, while the *y*-axis indicates the cumulative survival probability. Statistical comparisons between groups were performed using the log-rank test. Red: early surgery; Blue: late surgery.

**Figure 3:**
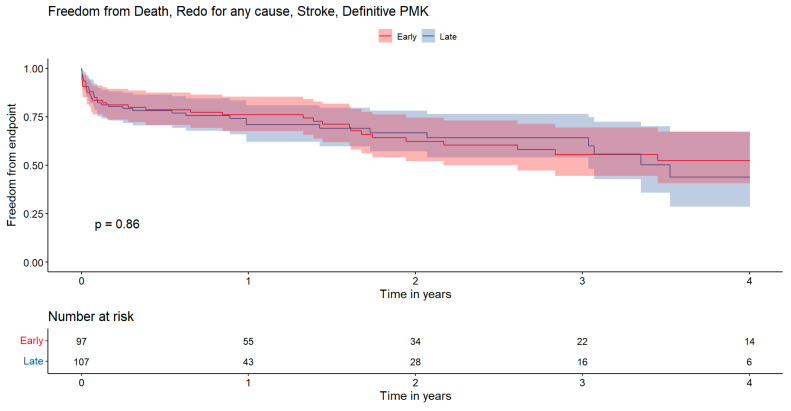
Kaplan–Meier survival curves illustrating incidence of composite endpoint including death, reintervention for any cause, stroke, and permanent pacemaker. The *x*-axis represents the time in years, while the *y*-axis indicates the cumulative freedom from events probability. Statistical comparisons between groups were performed using the log-rank test. Red: early surgery; Blue: late surgery.

Similarly, Figure 3 illustrates the 4-year composite endpoint with rates of 52.3 ± 13.1% for the EARLY group and 43.8 ± 18.6% for the LATE group (*p* = 0.86).

### 3.6. Logistic Regression

Table 6 presents the results of a univariate logistic regression analysis evaluating covariates associated with one-year mortality. Several factors showed significant associations with mortality. Chronic kidney disease (CKD) had an odds ratio of 2.62 (*p* = 0.01), and annular abscesses were also significantly associated with increased mortality (OR = 2.49, *p* = 0.01). ECMO use (OR = 16.56, *p* < 0.001) and prolonged mechanical ventilation (OR = 7.35, *p* < 0.001) were strongly correlated with higher mortality. Other factors, such as CPOD, large vegetation, and Staphylococcus Aureus, approached significance but did not reach a threshold (*p* = 0.06, *p* = 0.08, *p* = 0.09, respectively). Notably, the timing of surgery (early vs. late) did not significantly predict mortality at 1 year.

## 4. Discussion

This study sought to examine the clinical outcomes of patients with aortic infective endocarditis who were not in a condition requiring emergency surgery. These patients could undergo one of two timing strategies: early surgery (≤7 days) or late surgery (>7 days). The main findings are as follows:

- In-hospital mortality was not influenced by timing, with both early and late surgery groups showing a mortality rate of 16%.
- A higher incidence of permanent pacemaker implantation was observed in patients who experienced delays before surgery.
- Early surgery after a short course of antibiotics is sufficient for local sterilization and does not increase the risk of endocarditis recurrence.
- Predictors of adverse outcomes at 1 year after surgery include preoperative chronic kidney disease, presence of annular abscess, postoperative ECMO, and prolonged mechanical assisted ventilation. Additionally, PCR values and cardiopulmonary bypass duration are also linked to adverse outcomes, while factors such as surgical timing do not affect survival.

The general principle of infective endocarditis surgery is to achieve radical debridement of vegetation and infected tissue while avoiding or limiting, when possible, the use of prosthetic material. In cases of infective endocarditis involving native heart valves, the mitral valve (MV) is the most commonly affected, accounting for approximately 40–50% of cases [7]. The tendency to postpone surgery for stable patients with MV endocarditis can help achieve a high repair rate; however, at 10 years, overall survival is comparable between patients who undergo mitral valve replacement and those who undergo mitral valve repair [8].

The aortic valve is affected in 35–39% of cases [7]. Unlike mitral valve diseases, the potential for aortic valve repair is limited and technically challenging, with only 2.3% of patients undergoing aortic valve repair according to the EURO-ENDO registry [9]. In our series, no valve-sparing procedure was reported in both groups, likely due to the severity and complications present (e.g., large vegetations, perforations, abscesses). Perivalvular extension of IE is common, occurring in 10–40% of native valve cases and 56–100% of prosthetic valve cases, making it the leading cause of uncontrolled infection. The success of conservative treatment for perivalvular abscesses is low. Medical therapy alone is usually insufficient for local infection control, typically requiring surgical debridement and repair. In the present study, isolated aortic valve replacement (AVR), with or without patch reconstruction, was performed in 92% of cases. Thus, the need for more complex techniques remains limited to a small percentage of patients.

Lalani et al. demonstrated improved overall survival in patients undergoing surgery both in native and prosthetic valve infective endocarditis (IE) [10]. Thus surgical intervention is performed in 40–50% of infective endocarditis during the primary hospitalization [3]. However, the timing of surgery remains a matter of debate. Patients who do not undergo surgery within the first few days or week are at risk of developing complications that could ultimately render the procedure unfeasible. In spite of this, concerns about early surgery were the high mortality rates related to infectious state and scarce local tissue sterilization with risk of early reinfection. In our analysis, patients in the EARLY group (≤7 days) experienced the same mortality rate compared to those in the LATE group.

This finding adds to a highly heterogeneous body of literature. Some studies encourage early surgery [11,12], while others support a delayed approach [13,14]. The only randomized study on this topic is Kang’s 2012 trial, but the very small sample size, with only 76 patients, limits the ability to draw general conclusions [15].

Several factors influenced the planning of the treatment strategy. For instance, heart failure at admission plays a crucial role. The ICE-PCS study showed that patients with IE in NYHA class I-II had a surgical mortality rate of 7.9%, compared to 15% with medical therapy alone. This difference was even more striking in NYHA class III-IV patients, where surgical mortality was 23.4%, while medical therapy alone resulted in a mortality rate of 54.5% [16]. In our cohort, the prevalence of higher NYHA functional classes (III-IV) was comparable between groups (EARLY 36% vs. LATE 26%, *p* = 0.13).

Other factors must be taken into consideration in the decision-making process. Endocarditic lesions have may have different degrees of local invasiveness, leading to damage of the fibrous skeleton of the heart and conduction system and development of fistulas between adjacent cardiac chambers.

One finding of particular interest of this study is the trend towards a higher incidence of permanent pacemaker implantation in LATE group patients (EARLY 8.2% vs. LATE 16%, *p* = 0.100). We may hypothesize that delayed surgery may increase the risk of infection spread and antibiotic-induced tissue remodeling, leading to perivalvular fibrosis and greater susceptibility to intraoperative damage. A total of 15 patients with preoperative conduction disturbances were identified: six in the early group and nine in the late group, reflecting an increase of 33% of risk of developing a permanent AV block.

As a matter of fact, Hill and colleagues reported that the presence of preoperative conduction abnormalities together with *S. aureus* infection, annular abscess, tricuspid valve involvement, and prior valvular surgery are strong predictors of postoperative permanent pacemaker placement in infective endocarditis [17].

Another key argument for delaying surgery after antibiotic therapy completion is the belief that it improves local infection control and reduces IE recurrence. To test this hypothesis, we investigated whether early surgery might be associated with a higher incidence of re-endocarditis, either as reinfections or requiring repeat surgical interventions.

From our analysis, 5.3% of patients underwent reintervention for recurrence (EARLY: 6.2% vs. LATE: 4.4%, *p* = 0.7), while 7% experienced reinfection without reintervention (EARLY: 7.4% vs. LATE 6.7%, *p* = 0.8), without significant differences between groups. Our findings suggest that performing surgery after a short course of antibiotics does not increase the risk of endocarditis recurrence. Evidence in the literature was not univocal on this topic. The reported findings were in contrast with the results of the study by Thuny, reporting that early surgery (<7 days) may improve survival but entails a greater risk of relapses and valvular dysfunction [18]. Similarly, a recent meta-analysis noted a trend toward a higher recurrence rate of endocarditis in the early surgical group, though without statistical significance [12].

Recurrence, whether requiring reintervention or not, is a crucial outcome to consider. The epidemiology of infective endocarditis is evolving, with a rising incidence of prosthetic valve (PV) endocarditis. This trend is evident from comparisons of registries over the years. For example, PV IE now accounts for 30% of cases in the EURO-ENDO registry [9], which is an extremely high percentage when you consider that we started from 16% of cases in the 2008 French registry [19].

Prosthetic valve endocarditis is a significant complication of valve replacement, occurring at a rate of 0.3% to 1.2% per patient-year. Within the first five years post-implantation, approximately 3% to 6% of prosthetic valve recipients develop this complication [20].

If not addressed promptly, PV IE can result in an invasive infection of perivalvular structures resulting in a higher surgical mortality. Della Corte et al. documented 30-day mortality rates of approximately 20% in patients undergoing surgery for PV IE across three distinct eras. Similarly, the French experience, involving 54 patients who underwent reoperation for aortic re-endocarditis, reported a 30-day mortality rate of 18.5%, with a 5-year overall survival rate of 58.3 ± 18.6% and freedom from major adverse cardiac and cerebrovascular events (MACCEs) at 41.7 ± 19.7%. These findings underscore the relatively acceptable outcomes while also emphasizing the complexities and challenges of surgical management [21].

Finally, we sought to identify the strongest predictors of one-year mortality following cardiac surgery through a regression analysis. Predictors of adverse outcomes include preoperative chronic kidney disease, annular abscess, postoperative ECMO, and prolonged mechanical assisted ventilation. Additionally, the inflammatory state (CRP) and cardiopulmonary bypass duration are also linked to adverse outcomes, while surgical timing does not affect survival.

These results are aligned with those of a systematic review focusing on prediction models for postoperative mortality, which identified 11 preoperative factors associated with increased postoperative mortality. These factors include cardiogenic shock, NYHA class ≥ III, urgent surgery, paravalvular abscess, preoperative renal failure, previous cardiac surgery, specific etiologies (*S. aureus* and fungi), female sex, age, prosthetic valve IE, and multivalvular involvement [22].

### Limitations

The study has several limitations. First, there is no information available on patients who, after not undergoing surgery within the first 7 days, experienced complications such as hemorrhagic stroke that subsequently contraindicated the procedure. Additionally, the mean follow-up period of 637 days allows for insights into short- and medium-term outcomes but does not provide sufficient data to draw conclusions regarding the long-term outcomes of these patients. Finally, the data were collected from two centers which, despite having similar surgical procedure (namely, same % of isolated aortic valve replacement, Bentall, etc.) and comparable mortality rates, adopt different approaches: the French center tends to be more aggressive and contributed the majority of patients to the EARLY group, while the Italian center follows a more cautious strategy, typically aiming to administer at least 7 days of antibiotic therapy whenever possible before proceeding with surgery.

## 5. Conclusions

In conclusion, this study demonstrates that in patients with aortic infective endocarditis who can safely wait (i.e., do not require emergency surgery), a brief course of antibiotics is sufficient to ensure successful surgical outcomes, acceptable in-hospital mortality rates, and a life with a low risk of recurrence of infective endocarditis. Conversely, a watchful waiting approach does not provide advantages in terms of freedom from recurrence but exposes the patient to a potential risk of complications related to infective endocarditis during the prolonged duration of treatment.

## Figures and Tables

**Table 1:** Baseline and clinical characteristics.

	Overall (204 pts)	Early (97 pts)	Late (107 pts)	*p*-Value
Delay, days	8.0 [3.0–16.0]	3.0 [2.0–4.0]	15.0 [10.0–25.5]	<0.001
Age, years old	68.3 [60.0–75.0]	67.0 [58.0–75.0]	69.0 [62.0–75.0]	0.3
Gender, male	169 (83%)	79 (81%)	90 (84%)	0.6
BSA, m^2^	1.9 ± 0.2	1.9 ± 0.2	1.9 ± 0.2	0.7
BMI, Kg/m^2^	26.5 ± 5.6	26.6 ± 5.7	26.4 ± 5.5	0.9
Drug abuser	4 (2.0%)	1 (1.0%)	3 (2.8%)	0.6
Hypertension	121 (59%)	53 (55%)	68 (64%)	0.2
CPOD	15 (7.4%)	6 (6.2%)	9 (8.4%)	0.5
CKD	40 (20%)	17 (18%)	23 (21%)	0.5
Dialysis	5 (2.5%)	3 (3.1%)	2 (1.9%)	0.7
NYHA III-IV	63 (31%)	35 (36%)	28 (26%)	0.13
Nosocomial Infection	5 (2.5%)	2 (2.1%)	3 (2.8%)	>0.9
Fever	160 (78%)	74 (76%)	86 (80%)	0.5
Cardiogenic Shock	10 (4.9%)	5 (5.2%)	5 (4.7%)	>0.9
Previous CVE	56 (27%)	25 (26%)	31 (29%)	0.6
Splenic Abscess	33 (16%)	17 (18%)	16 (15%)	0.6

BMI: body mass index; BSA: body surface area; CKD: chronic kidney disease (defined as estimated glomerular filtration rate < 50 mL/min/1.73 m^2^); CPOD: chronic pulmonary obstructive disease; CVE: cerebrovascular event; NYHA: New York Heart Association; Pts: patients. Values are expressed as n (%), mean ± standard deviation, median [quartile 1–quartile 3].

**Table 2:** Preoperative echocardiographic and biological parameters.

	Overall (204 pts)	Early (97 pts)	Late (107 pts)	*p*-Value
AV prosthesis	65 (32%)	26 (27%)	39 (36%)	0.14
Mitral valve involvement	53 (26%)	30 (31%)	23 (21%)	0.12
Large Vegetation *	165 (81%)	85 (88%)	80 (75%)	0.02
Annular Abscess	76 (37%)	34 (35%)	42 (39%)	0.5
Fistula	8 (3.9%)	4 (4.1%)	4 (3.7%)	>0.9
LVEF	57.0 [55.0–64.0]	55.0 [53.0–63.0]	58.0 [55.0–64.0]	0.4
sPAP	30.0 [28.0–32.3]	30.0 [28.0–31.0]	30.0 [28.0–33.0]	0.7
Haemoglobin, g/dL	10.5 [9.3–11.8]	10.6 [9.4–11.6]	10.3 [9.3–11.9]	0.6
White Blood Cells,·1/µL	9.6 [6.7–12.2]	10.2 [7.7–12.5]	8.7 [6.4–11.7]	0.049
Serum Albumin Level, g/dL	2.8 [2.4–3.3]	2.7 [2.4–3.1]	2.9 [2.4–3.4]	0.4
Peak C-Reactive Protein Level, ng/dL	94.8 [55.5–145.3]	93.0 [59.5–152.2]	95.0 [44.6–133.7]	0.2

LVEF: left ventricular ejection fraction; sPAP: systolic pulmonary arterial pressure; Pts: patients. * Large vegetations are defined as those measuring ≥ 10 mm on echocardiography. Values are expressed as n (%) or median [quartile 1–quartile 3].

**Table 3:** Intraoperative data.

	Overall (204 pts)	Early (97 pts)	Late (107 pts)	*p*-Value
Biological AVR	178 (87%)	87 (90%)	91 (85%)	0.3
Mechanical AVR	9 (4.4%)	5 (5.2%)	4 (3.7%)	0.7
Homograft	12 (5.9%)	3 (3.1%)	9 (8.4%)	0.11
Root/Bentall surgery	14 (6.9%)	7 (7.2%)	7 (6.5%)	0.8
Associated CABG	13 (6.4%)	6 (6.2%)	7 (6.5%)	>0.9
Associated Mitral surgery	50 (25%)	25 (26%)	25 (23%)	0.7
Other associated procedures *	22 (11%)	9 (9.3%)	13 (12%)	0.5
CPB Time, min	134.5 [97.8–194.3]	124.0 [97.0–196.0]	140.0 [99.5–192.0]	0.5
Aortic CC Time, min	107.0 [80.0–149.5]	104.0 [77.0–149.0]	109.0 [82.0–149.5]	0.5

AVR: aortic valve placement; CABG: coronary artery bypass grafting; CC: cross-clamping; CPB: cardiopulmonary bypass; Pts: patients. * Associated procedures: left appendage closure, tricuspid surgery, atrial fibrillation ablation, pacemaker implantation. Values are expressed as n (%) or median [quartile 1–quartile 3].

**Table 4:** Early outcomes (in-hospital complications).

	Overall (204 pts)	Early (97 pts)	Late (107 pts)	*p*-Value
IABP	11 (5.4%)	3 (3.1%)	8 (7.5%)	0.2
ECMO	13 (6.4%)	7 (7.2%)	6 (5.6%)	0.6
MAV > 48 h	33 (16%)	14 (14%)	19 (18%)	0.5
Bleeding	6 (2.9%)	4 (4.1%)	2 (1.9%)	0.4
Sternal Wound Infections	8 (3.9%)	4 (4.1%)	4 (3.7%)	>0.9
Postoperative AF	62 (30%)	23 (24%)	39 (36%)	0.048
Postoperative Stroke	3 (1.5%)	1 (1.0%)	2 (1.9%)	>0.9
KDIGO				0.6
No Renal impairment	141 (69%)	67 (69%)	74 (69%)	
Stage 1	21 (10%)	11 (11%)	10 (9.3%)	
Stage 2	22 (11%)	8 (8.2%)	14 (13%)	
Stage 3	20 (9.8%)	11 (11%)	9 (8.4%)	
Definitive PMK	25 (12%)	8 (8.2%)	17 (16%)	0.10
Hospital length of stay	18.0 [12.0–27.3]	18.0 [12.0–26.0]	19.0 [10.5–28.5]	0.8
In-Hospital Death	33 (16%)	16 (16%)	17 (16%)	>0.9
Death at 1 Year	42 (21%)	19 (20%)	23 (21%)	0.7

AF: atrial fibrillation; ECMO: extracorporeal membrane oxygenation; IABP: intra-aortic balloon pump; KDIGO: kidney disease improving global outcomes; MAV > 48 h: mechanical ventilation > 48 h; PMK: permanent pacemaker; Pts: patients. Values are expressed as n (%) or median [quartile 1–quartile 3].

**Table 5:** Long-term outcomes (out-hospital complications).

	Overall (171 pts)	Early (81 pts)	Late (90 pts)	*p*-Value
Any kind of IE (operated and not operated)	21 (12%)	11 (14%)	10 (11%)	0.6
Not operated IE relapse	12 (7.0%)	6 (7.4%)	6 (6.7%)	0.8
Redo for IE relapse	9 (5.3%)	5 (6.2%)	4 (4.4%)	0.7
New CVE	5 (2.9%)	2 (2.5%)	3 (3.3%)	>0.9
Death at last follow-up	28 (16%)	15 (19%)	13 (14%)	0.5

CVE: cerebrovascular events; IE: infective endocarditis; Pts: patients; Redo: surgical reintervention. Values are expressed as n (%). Patients who died during the main hospitalization were excluded.

**Table 6:** Univariate logistic regression.

Covariate	Odds Ratio	95%CI Lower	95%CI Upper	*p*-Value
Timing (early surgery)	1.12	0.57	2.24	0.74
Age	1.02	1.00	1.06	0.12
Gender (male)	0.70	0.31	1.71	0.41
BSA	0.96	0.20	4.43	0.96
CPOD	2.83	0.90	8.38	0.06
CKD	2.62	1.20	5.60	0.01
AV prosthesis	1.24	0.60	2.52	0.55
Cardiogenic Shock	1.70	0.35	6.44	0.46
Large Vegetation	0.50	0.23	1.12	0.08
Annular Abscess	2.49	1.25	5.01	0.01
Multiple Valve Involvement	0.99	0.41	2.19	0.98
Pulmonary Hypertension	1.91	0.85	4.14	0.10
CVE	1.24	0.58	2.57	0.57
Splenic Abscess	1.05	0.39	2.50	0.92
Staphylococcus Aureus	2.10	0.87	4.81	0.09
PCR	1.00	1.00	1.01	0.02
CPB	1.01	1.00	1.01	0.00
ECMO	16.56	4.77	76.99	0.00
MAV > 48 h	7.35	3.29	16.78	0.00
KDIGO	—	—		
Stage 1	1.60	0.49	4.57	0.4
Stage 2	2.39	0.84	6.38	0.088
Stage 3	2.76	0.95	7.54	0.051
Definitive PMK	0.30	0.05	1.08	0.11

Covariate correlates to mortality at 1 year. Acronyms are previous described.

## Data Availability

The raw data supporting the conclusions of this article will be made available by the authors on request.

## Abbreviations

The following abbreviations are used in this manuscript:

AF	Atrial Fibrillation
AVR	Aortic Valve Replacement
BMI	Body Mass Index
BSA	Body Surface Area
CABG	Coronary Artery Bypass Grafting
CC	Cross-Clamping
CKD	Chronic Kidney Disease
CPB	Cardiopulmonary Bypass
CPOD	Chronic Pulmonary Obstructive Disease
CRP	C-Reactive Protein
CVE	Cerebrovascular Event
ECMO	Extracorporeal Membrane Oxygenation
ESC	European Society of Cardiology
IE	Infective Endocarditis
IABP	Intra-Aortic Balloon Pump
KDIGO	Kidney Disease Improving Global Outcomes
LVEF	Left Ventricular Ejection Fraction
MACCEs	Major Adverse Cardiac and Cerebrovascular Events
MAV	Mechanical Assisted Ventilation
MRI	Magnetic Resonance Imaging
NYHA	New York Heart Association
PET	Positron Emission Tomography
PMK	Permanent Pacemaker
PV	Prosthetic Valve
sPAP	Systolic Pulmonary Arterial Pressure
